# A qualitative study of Chinese Canadian fathers’ smoking behaviors: intersecting cultures and masculinities

**DOI:** 10.1186/s12889-015-1646-0

**Published:** 2015-03-25

**Authors:** Aimei Mao, Joan L Bottorff, John L Oliffe, Gayl Sarbit, Mary T Kelly

**Affiliations:** Kiang Wu Nursing College of Macau, Est. Repouso No.35, R/C, Macau, China; School of Nursing and Institute for Healthy Living and Chronic Disease Prevention, University of British Columbia, 3333 University Way, Kelowna, BC Canada; Faculty of Health Sciences, Australian Catholic University, Melbourne, VIC Australia; School of Nursing, University of British Columbia, 302 6190 Agronomy Rd, Vancouver, BC Canada; Institute for Healthy Living and Chronic Disease Prevention, University of British Columbia, 3333 University Way, Kelowna, BC Canada

## Abstract

**Background:**

China is home to the largest number of smokers in the world; more than half of the male population smoke. Given the high rates of Chinese immigration to Canada and the USA, researchers have explored the effect of immigration on Chinese smokers. Reduced tobacco use among Chinese immigrants has been reported in the United States; however, little is known about the social factors underlying men’s smoking practices in settings where tobacco control measures have denormalized smoking, and in the context of fatherhood. The purpose of this Canada-based study was to explore the smoking-related experiences of immigrant Chinese fathers.

**Methods:**

In this qualitative study, semi-structured telephone interviews were conducted with 22 Chinese Canadian fathers who smoked or had recently quit smoking, and had at least one child under the age of five years old.

**Results:**

The Chinese fathers had dramatically changed their smoking patterns due to concern for their children’s health and social norms and restrictions related to smoking in Canada. The facilitators and barriers for men’s smoking were intertwined with idealized masculine provider and protector roles, and diverse Canadian Chinese cultural norms related to tobacco use.

****Conclusion**s:**

The findings have implications for the development of future smoking cessation interventions targeting Chinese Canadian immigrant smokers as well as smokers in China.

## Background

China has the largest number of tobacco smokers in the world and the majority of smokers are men [1]. Survey data in China indicates the extent to which smoking is a gendered health concern: 52.9% of adult men smoke, compared to 2.4% of adult women [1]. As a culturally accepted practice among men, and conduit for interacting socially in China [2-4], the predictors of tobacco use include: being male, being employed, married, and having less than high school education [5].

In the US and Canada, a large and growing proportion of immigrants are from China. Chinese Canadians are the second largest visible minority group in Canada, comprising 4% of the total population, and over 70% are foreign born [6]. In the US, Asian Americans represent the second largest immigrant group, and among foreign born Asians, 23% are Chinese, comprising more than 2.5 million people [7]. There is evidence that immigration may influence reductions in smoking because the rate of smoking prevalence among Chinese immigrants appears to be much lower than their counterparts in China [8-11]. For instance, in the U.S. the smoking prevalence among recent immigrant men from China (<5 years in the U.S.) was 28.0% [10] although that is still higher than the smoking prevalence in the general American population (18.1%) [12]. Studies exploring Chinese men’s smoking patterns in different parts of America provide the following prevalence rates: 22% in Seattle [9]; 16.1% in Texas [10]; 24% in Delaware Valley and New Jersey [13]; 29% in New York City [14] and 16.2% [15] based on national survey data. Despite these favorable trends suggesting reduced smoking rates post immigration, nearly two thirds of male Chinese immigrants who are current smokers have no intentions to quit smoking in near future [13]. Little research is available regarding smoking prevalence among Chinese Canadian immigrants.

In comparison, within Canada, 21.8% of young men aged 25 to 34 years are current smokers [16]. Similarly, in the US, 26.8% of men aged 25–44 are smokers, the highest consumption category aggregated by age and sex [12]. In China, the rate of smoking prevalence for men aged 24–44 years reaches 59.3% [1]. It is noteworthy that these age ranges also represent the stage of life at which men become fathers. As such, although immigrant Chinese men enter a society in which tobacco use is denormalized, their Canadian and American male peers are modeling higher than average levels of tobacco consumption (i.e., 16% of the Canadian adult population are current smokers [16]), further challenging Chinese men’s motivations to reduce their tobacco use after immigration.

### Tobacco use, culture and masculinities

Research from different cultures has revealed that tobacco use is universally associated with gender-related factors. For example, across Asian countries, including China, men’s smoking is socially accepted, while women’s smoking is discouraged; in contrast, in the Western world, women smoke at almost the same rate as men [17]. Despite these significant differences in smoking prevalence between men and women in Asian countries, little research has explored gender-related factors underlying these smoking patterns. Research studies in the West have shown that men’s smoking is closely related to masculine ideals, such as independence, physical resilience to harmful substances and capacity to endure risk-taking [18-21], while women’s smoking symbolizes personal freedom, sexual attraction, and emancipation from gender norms [22,23].

In China, smoking has provided a strong positive signifier of masculinity and an important way to embody idealized notions of manhood and enact authority [24]. As a culturally accepted practice, smoking is closely related to social currency and masculine capital [2,3,5]. As such, smoking and cigarette gifting are an important component of men’s social interactions and business transactions, even among those residing in rural areas [3,4,25], and these gendered practices challenge tobacco reduction efforts in China [26].

Despite men’s reliance on smoking to fulfill gendered roles, there is some evidence that Chinese men are willing to decrease their smoking in the context of particular lifecourse events. For example, in one study it was reported that the prevalence of second-hand smoke exposure was 55.9% among women before pregnancy and decreased to 41.9% during pregnancy [27]. Among husbands who ever smoked, 14.4% stopped smoking before pregnancy, 38.1% changed their smoking behaviors during pregnancy, and 10.7% quit smoking after pregnancy [27]. However, men’s changes in smoking behavior during pregnancy were temporary, and most fathers started smoking again by the time their children turned two years old [28,29]. It has been suggested that changes in health-related behavior associated with becoming a father may be related to men’s fulfilment of socially subscribed expectations to meet the needs of a dependent [30]. Despite this, among Chinese men it appears that the functions that smoking serves in social and economic encounters and limited awareness of its health consequences [25] may create a compelling context to resume smoking to maintain traditional breadwinner roles and social status in an environment where the majority of men smoke.

In Canada, there is also emerging evidence that fatherhood may be associated with changes in smoking patterns. Qualitative interviews with fathers who smoke have revealed that engagement in fathering often heightens dissonance regarding continued smoking; as men’s alignment with masculinities shifts to being protectors and providers, they seriously consider and sometimes try to quit smoking [31-33]. These shifts need to be understood in the context of tobacco control policies in Canada which have significantly reduced smoking levels, and created a social environment where smoking is denormalized and increasingly stigmatized [31,34]. This, along with the prominence of tobacco control messages about the health effects of smoking, challenges men’s continued smoking as they incorporate contemporary identities associated with fathering [31]. Following immigration, Chinese men are exposed to new social environments in their host countries that may influence their smoking practices. Li [35] has observed that despite some reductions in smoking, there appears to be an enduring influence of the Chinese smoking culture on Chinese immigrant smoking. However, how changes in social environments associated with immigration play a role in shaping Chinese father’s smoking is not entirely clear. The purpose of this Canada-based study was to describe the intersections between cultures and masculinities among Chinese Canadian immigrant fathers who smoke.

## Methods

This study was conducted using a qualitative interpretive approach, guided by grounded theory methods [36]. Men were recruited if they met the following criteria: 1) self-identified as a Chinese immigrant or a Chinese Canadian; 2) were expecting a child or had a child under five years old; 3) were currently smoking or had quit smoking in the past 5 years; and 4) had lived in Canada for at least 6 months. Bilingual recruitment ads were distributed to Chinese organizations in the lower mainland of British Columbia, Canada and posted on Chinese online forums. Among the 22 Chinese fathers recruited, 12 resided in Ontario, two in Quebec, and eight in British Columbia, representing the three most populated provinces in Canada. The sample was characterized by a variety of backgrounds in terms of demography and smoking patterns (Table 1). All the fathers were first-generation immigrants; two migrated to Canada with their parents before 18 years of age, and the other 20 migrated after age 18. The two early immigrants identified their first language as English; the others identified Chinese as their first language.

**Table 1:** **Demographics and smoking history of the participants**

**Age (years)**	**37.8±5.2 (28 to 46)**
**Education**	
Junior/middle school	1
High school	0
Non-university (collage, vocational, technical, trade etc.)	3
Bachelor’s degree	13
Master’s degree or over	5
**Years in Canada**	**6.8±4.4 (0.5 to 13)**
**Occupation**	
Clerical/administrative	5
Construction/manual labour	7
Technical/skilled/professional/trade	7
Unemployed (student)	3
**Marital status**	
Married	21
Divorced	1
**Number of children**	
1	13
2	8
3	1
**Amount smoked**	
<10/day	7
10-20/day	3
>20/day	0
Quit smoking	12

The study was reviewed and approved by the University of British Columbia Behavioral Research Ethics Board. All the participants provided informed consent. They were offered an honorarium of CAD$50 to acknowledge their contribution to the study.

### Data collection

Semi-structured interviews were conducted via telephone with all the participants except one, with whom a face to face interview was conducted. Canada is a vast country in terms of geographical size and telephone interviewing maximized our ability to reach Chinese immigrants from diverse backgrounds. Also, telephone interviewing is ideal to explore sensitive issues because it permits more anonymity and privacy than face to face interviewing [37]. The interview questions focused on experiences of smoking in the context of fathering and men’s efforts to quit smoking (Table 2). Probes and follow-up questions were used to encourage the fathers to elaborate on their smoking patterns before and after they came to Canada, and how their smoking had changed around their partners’ pregnancies. A brief questionnaire was used before the start of the interview to collect basic demographic information and smoking patterns. The interviews were conducted by a bilingual researcher (first author). Two of the interviews were conducted in English while the other 20 were in Mandarin. The interviews lasted from half an hour to 1.5 hours, and on average were one hour.

**Table 2:** **Interview questions**

**Topic areas**	**Questions**
**Experiences in fathering**	Tell me a little about your family.
	• Tell me about your experience when you first became a father.
	• What kind of father do you think is a good father?
	• How have things changed (at home, at work) since you have had a child?
	Who looks after your child/ren?
	• How are you involved in looking after your child/ren?
**Experiences with smoking**	Tell me about your smoking.
	➢ How is your smoking at home? Are there any rules on smoking in your home?
	➢ How is your smoking in your car? At work?
	➢ How has becoming a dad changed your smoking?
	What helped you the most to quit smoking? What were the barriers?
	How are Chinese fathers different from the Canadian fathers in terms of smoking practices? What do you think causes the differences?

### Data analysis

All interviews were digitally recorded, translated into English and transcribed. A bilingual research assistant with Chinese and English proficiency translated the interviews and the translations were checked by the bilingual researcher (first author). An interpretive thematic analysis using constant comparison was conducted with transcribed interviews [36]. A coding framework was developed by the research team based on their previous experiences of research with Canadian fathers who smoked and the readings of the first three interviews. Three members of the research team independently hand-coded these three interviews. Definitions for the codes were established to facilitate coding. After comparisons were made, the researchers refined the coding framework. Once the coding framework was finalized, the qualitative data management program NVIVO 8 was used to code and retrieve data. Data coded to each category were reviewed in detail, comparing and contrasting data from all participants to identify patterns in the Chinese Canadian immigrants’ constructions of their smoking under the context of being fathers. The two participants who had migrated to Canada as children were compared and contrasted with other participants who migrated as young adults to ensure they were not outliers. The research team reviewed and debated discordant narratives to reach consensus on the interpretation of the findings. Repeated narratives about men’s smoking were evident and indicated data saturation was achieved.

## Results

Twelve of the fathers had quit smoking (defined as having stopped smoking for at least a week); 10 fathers were currently smoking, including the two participants who had migrated at a younger age. All the fathers had experienced significant changes to their smoking related to two life changes: immigration and becoming a father. Although only one Chinese father quit smoking soon after he moved to Canada, the majority of the others reduced the numbers of cigarettes smoked after immigrating. The men described becoming a father as a more influential factor than immigration, and one that led to more quits and more significant smoking reductions.

Participants had developed the habit of smoking outside the home after they arrived in Canada, and the home smoking ban was more strictly abided by the fathers during their partner’s pregnancy and the early childhood years. Despite reduced levels of smoking, the 7 current light smokers (<6–7 cigarettes a day) were vague about their commitment to reducing or quitting their last few cigarettes.

**Figure 1:**
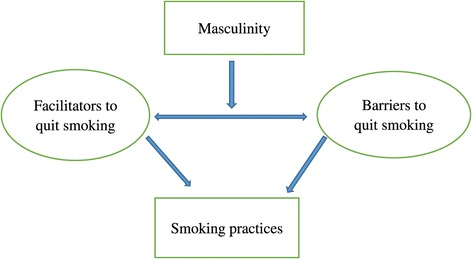
**The impacts of masculinity on smoking practices.**

A variety of factors contributed to smoking cessation or continued smoking, as summarized in Table 3. These factors were strongly influenced by and linked to the masculine ideals of fatherhood as a provider and protector of children and family. Masculine ideals and behaviors displaying masculinity are socially constructed, multifaceted, and dependent on specific and local social contexts [38] and, therefore, shaped the facilitators and barriers related to the fathers’ smoking practices in diverse ways (Figure 1). In the following section, the relationship between smoking, fathering and masculinity for Chinese Canadian immigrant fathers is detailed.

**Table 3:** **Facilitators and barriers to quitting smoking**

**Facilitators**	**N**	**Barriers**	**N**
Concern over impacts of smoking on children’s health	22	Light smoking	10
Economic concern	20	Refusal of use of smoking cessation aids	9
Different smoking environment between China and Canada	22	Having friends or colleagues who smoke	7
Smoking cessation supports from wives and other people	14	Difficulty in getting rid of the habit	6
Smoking cessation supports from health professionals	8	The need for coping stress, and killing time	8
Concern over impacts of smoking on own health	11

### The masculine identity of protector and smoking

Becoming a father was considered the most important facilitator for quitting or reducing smoking. Eleven of the 12 ex-smokers quit smoking when they learned that they were going to have children or when their children were in infancy. Others had substantially reduced their smoking for their expected child or young children.

According to the fathers, the message that exposure to second-hand smoke is harmful to pregnant women and young children had become commonplace. A 46-year-old father who had quit smoking said, “I can’t imagine that there are men who still smoke around their wives during pregnancy.” Men’s changes in smoking were constructed as voluntary behavior modifications, rather than forced practices.

Usually Chinese smokers developed a habit of smoking outside the home as soon as they arrived in Canada, because smoking is not permitted inside public buildings. A 33-year-old father, smoking 3–4 cigarettes/day, said: “We could smoke everywhere in China. Here no one smokes inside buildings. Under such circumstances, we smoke much less than we did in China.” The Chinese men were willing to conform to Canadian smoking norms, and extended the ban on indoor smoking in the public sphere into domestic spaces. Also, becoming a father strengthened efforts to maintain a smoke-free home. For example, the majority of fathers stated they made no exceptions to keeping their smoking outside, even during the cold Canadian winter months.

The no-indoor smoking rule also applied to visitors in their homes. A 33-year-old father who currently smoked 15 cigarettes daily spoke of the rule in his home: “Here I have some friends who smoke. When they come to my home they don’t smoke inside. We all go outside to smoke. You don’t have to tell them to do so. That’s a rule here”.

This father’s narrative fulfills several purposes. Demonstrated is his knowledge of Canadian cultural norms and the stigma related to indoor smoking. Further, in emphasizing how he and his friends respected these “rules”, his concession also operates as a rationalization for his own continued outdoor smoking. It is implicit that the “we” in his interview refers to male friends who enjoy smoking together and that they have adjusted their smoking practices to reflect Canadian values.

Involvement in childcare also increased the Chinese fathers’ determination to restrict their smoking at home. A 40-year-old father who smoked fewer than 10 cigarettes/week, said it was self-evident that more involvement in childcare would reduce smoking: “During the time you are with your baby, you will be too busy to smoke.” The fathers offered two reasons why Chinese Canadian fathers spent more time with their children than their counterparts did in China. One reason related to the observation that, in Canada, fathers tend to share childcare tasks with their wives; therefore, the Chinese 'Canadian fathers shifted their own behaviors to align with these contemporary domestic practices for Western men. Traditionally, there is a gendered division of family responsibilities in China: men deal with outside issues while women are responsible for domestic tasks and childcare. Although the Chinese Canadian fathers stated that the main caregivers of their children were their wives or partners, they acknowledged that their increased involvement in childcare in Canada also reflected their fewer social networks and financial resources.

In China, it is usually the grandparents who take care of their grandchildren. In Canada, we can’t get our parents to help with the childcare. It is very expensive to hire a babysitter too. A babysitter’s salary is almost the amount of my salary. [41-year- old father, smoking 2–3 cigarettes/day].

The other reason for increased childcare duties related to differences in men’s leisure activities between China and Canada. In China, spending time with friends, colleagues, and business partners was an important component of men’s pastime activities and a symbol of men’s social status. However, Chinese Canadian fathers observed a different use of leisure time by Canadian fathers, as a 44-year-old Chinese Canadian father who smoked 10 cigarettes/day professed:

In China, if a man spends much time at home he will be regarded by other people as lacking a well-established social network. Canadian fathers tend to stay at home or go on trips with their family on weekends. I am starting to develop the habit of travelling with my family now. This is something different from what I did in China, but I feel really good.

Like him, other Chinese fathers did not challenge Canadian fathers’ involvement in family life; instead they questioned the image of the traditional Chinese father as an “absent father”. This questioning touches on the fundamental contradiction of emphasized Chinese values of familism and the lack of childcare involvement that is often observed among Chinese fathers.

Despite reduced smoking, some fathers reported smoking throughout their partners’ pregnancies, although at a lighter level, fewer than 10 cigarettes a day. Several fathers who had quit smoking during their partners’ pregnancies resumed smoking postpartum. These fathers were able to reconcile their continued smoking with the role of protector. For example, a 44-year-old father, smoking 10 cigarettes/day, boasted that his three-year-old daughter did not know he was a smoker: “In the last years, I have never smoked at home. And I have always tried to hide my smoking from my kid. I don’t think she knows I am a smoker”. Implicit here is the man’s denial that his concealed smoking will directly (via second-hand smoke) or indirectly (as a role model) impact his child. For this reason, some of the current smokers were vague in their commitment to quitting their last few daily cigarettes. A 40-year-old father who reported smoking fewer than 5 cigarettes a day said: “To be honest, I have never thought to completely stop smoking one day, although that is the ideal”.

### The masculine identity of provider and smoking

In most of the participants’ families, the wives were either full-time caregivers or worked part-time, while the fathers, as primary breadwinners, worked full-time or had two part-time jobs. The career prospects of the Chinese men were negatively impacted as a result of immigration. Although the men were relatively well educated, the majority were unable to find suitable jobs that matched their career training. As a result, they were underemployed, working as labourers or in lower skilled jobs with poor salaries. Nonetheless, the Chinese fathers seemed to agree that the first-generation’s hardships and sacrifices were inevitable: “Why have you left your home town and come to a foreign country? There must be a reason. You come here to seek a better life, so you will have to invest in that”. (41-year-old, ex-smoker).

The cost of more expensive cigarettes in Canada added to the participants’ financial burdens. A 39-year-old father, smoking 1–3 cigarettes/day, made the comparison saying, “Generally a pack of cigarettes costs 10 dollars in Canada. The cheaper ones are 6–7 dollars. In China, a pack of cigarettes usually costs 10 RMB, which is less than 2 dollars”.

As providers for their families, the fathers also realized the importance of their own health. Although the fathers were generally healthy, they became nervous if they did not feel well. A 37-year-old father who smoked 6–7 cigarettes/day said, “You are the father; you are the supporter; you have to take care of your family. If you get cancer from smoking, then you will be a liability to your family!” The pressures of breadwinner status are apparent here; the fathers were acutely aware of how their own health was the prerequisite for a better future for their children.

Interestingly, the fathers who were currently light smokers (fewer than 10 cigarettes a day) were not concerned about the health risks of their smoking. They connected the idea of “light” smoking with the idea of natural balance, declaring their smoking harmless. One 33-year-old father, who currently smoked 3–4 cigarettes a day, said:

There are two types of views on quitting smoking. One said it is good to quit because it is better for the health. The other said that you shouldn’t do it too fast because your body has got used to nicotine. I agree with the second point. I suppose this reflects the Chinese culture of shun-qi-zi-ran [following a natural course without much change].

According to the fathers, their bodies had established a balance with smoking. The complete withdrawal from smoking could disrupt the balance and might cause health problems. This finding is in line with studies in China indicating that some health professionals have suggested smokers not quit completely or too suddenly [39,40]. This light cigarette smoking rationale might also be related to the popularity of the cigarettes marketed as “light” among Chinese people.

As the family provider, the fathers also justified their right to smoke. They expressed their loneliness in Canada, their stress associated with finding a job, and the frustration caused by their job. A 46-year-old father who smoked fewer than 10 cigarettes a week expressed difficulty in quitting, but defended his smoking. Based on this father’s presentation, his smoking did not tarnish his family man image, but rather, represented a hard-won reward:

My wife doesn’t say anything about my smoking. She knows I need it. I don’t feel uneasy with my smoking as long as I have earned enough for my family to buy food and clothes. To tell you the truth, I always give wife and two children the best and leave the worst to myself. My wife and my eldest daughter have one cell phone each and their cell phones are more advanced than mine.

Evident here is the alignment to well established norms around smoking and the self-sacrifice synonymous with masculine virtues providing for others. Ever clear are bi-cultural values whereby the man’s consumerism relied on and reflected a range of Chinese and Canadian cultural norms.

### Masculine identity as an autonomous man and smoking

Smoking, as a predominately male behavior, was reflected in that none of the participants’ partners were smokers. The men described how their partners held negative attitudes towards their smoking and often asked them to quit, which they acknowledged had played a role in their changed smoking to a certain extent. However, they emphasized that the changes in their smoking patterns were their own decisions rather than concessions to their partners. A 42-year-old father, who had quit smoking three years ago when he learned that his wife was pregnant, said: “I really think that this is something only depending on you. No matter how many people tell you to quit, they can’t be there to watch you 24 hours”. Two fathers quit smoking partially due to advice they received from physicians while accompanying their partners to a consult. Other fathers also added the importance of physicians in encouraging them to quit smoking, but the majority acknowledged that they did not seek or get help from health professionals.

All the fathers had made more than one quit attempt, and at the time of the interviews 12 fathers were smoke free. Participants, including the current smokers, expressed difficulty in quitting because of the habit of smoking, but downplayed the usefulness of cessation aids. They also had little interest in counseling or telephone services even if the services were free. A 28-year-old father who had quit smoking for about three months pointed out that Chinese men do not use smoking cessation services: “Canadians like to use the hotline but it doesn’t mean it is useful. Sometimes they just like to call to chat. Very seldom Chinese call it”. He attributed this indifference to cessation services to personal qualities of self-reliance and independence, lofty attributes cultivated in Chinese culture:

Canadian governments can help its people to solve problems. The governments in China don’t care about you; so who you can rely on if you don’t rely on yourself? Chinese people rely on themselves or their close friends when they come across problems. No matter how hard their life is, Chinese tend not to rely on governments.

The fathers insisted that a successful quit required decisiveness and willpower on their part, and that if a man “wanted to quit” nothing more was needed. They framed external aids, physical or psychological, as strategies that Caucasian men relied on. In this way, the Chinese men positioned themselves as more autonomous and stronger willed than Canadian men.

Smoking was also perceived as a personal hobby and the Chinese fathers tended not to share their smoking and quitting experiences in fear that their privacy would be violated. A 33-year-old father who had quit smoking expressed concern about losing face if he went to cessation services:

There are not a lot of Chinese here so everyone knows each other. Your privacy will lessen within the small group and your friends and relatives may know of that. You know, we Chinese care about our face. We do not want to give other people any bad image about us.

Saving face and the protection of privacy in this context can also be interpreted as a means of maintaining a public image of masculine strength, invulnerability, and being in control. Also evident in the commentaries is the existence of masculine hierarchies, whereby the participants jockeyed for position among Chinese immigrants, making assertions about Eastern and Western men’s legitimate purchase on hegemonic masculinity.

## Discussion

The findings from the current study provide important empirical and theory based insights into Chinese Canadian fathers’ smoking. The facilitators and barriers to participants’ smoking reflect findings drawn from previous studies chronicling how immigrants from cultures that normalize smoking tend to reduce their smoking after immigrating to countries that strive to be smoke free [41]. However, ever present in the current findings are complex connections underpinning Chinese Canadian fathers’ concessions around smoking post-immigration. For example, adherence to traditional Chinese values of familism and collectivism appear to cultivate efforts toward smoking cessation within the Canadian context. At the same time, Western norms around reducing smoking to benefit one’s health as well as aid family well-being also emerged as influencing men’s efforts to be smoke free.

Sociologists report that Chinese individuals tend to pursue the collective interests of the family rather than individual interests [42]. In the context of the current study, protecting children from the harms of tobacco smoke is normative, reflecting Chinese cultural ideals of familism. Although not overtly expressed, many participants suggested feeling pleased, and perhaps lucky that they were able to conform to Canadian cultural ideals about maintaining a smoke-free home. Reflected in this bicultural positioning is evidence of both acculturation and a keen sense of traditional Chinese values, including the need to save face in the host country. In terms of masculinities, this willingness to conform to Canadian values and tobacco use norms might indicate Chinese Canadian immigrant men harbored little interest in rebellion or protesting against dominant cultural ideals and structures by smoking in prohibited ways and/or places. In contrast, Chinese Canadian fathers self-sacrificed in a range of ways (e.g., underemployment, financial burden, isolation, etc.) to make good on the patriarchal promise of providing a better life for their families. It can also be reasonably argued that smoking in Canada was less focussed on being with and/or connecting with other men. In this respect there were likely fewer masculine pressures for men to smoke in Canada, especially given the absence of ritual cigarette sharing among men in the West. Despite upholding familism, Chinese fathers could be providers rather than involved caregivers due to the traditional cultural divisions of domestic and family care labor. An important change for the Chinese Canadian fathers was the increased caring responsibilities embodied as fathering norms in Canada. The direct childcare norms and expectations enhanced the fathers’ identity as protector of their children. Ultimately, this change contributed to their smoking reductions, because the more time fathers spent with their children, the less likely they were to smoke, a finding that is in line with other studies [33,43]. From the perspective of the Chinese Canadian fathers, their introduction to and the permission to be more involved in fathering– when taken up - was framed as a masculine virtue reflecting some Chinese and Canadian ideals. In this respect, being an engaged father reflected their alignments to a range of bicultural norms.

Masculine ideals of self-control and autonomy are regarded as important influences in men’s attempts to quit smoking [21,32,33]. The majority of men quit smoking without aids in all cultures [44]; however, more than 90% of Chinese smokers quit unaided [45], compared to two-thirds to three-quarters of smokers elsewhere [44]. Our study showed that Chinese men’s self-reliance in quitting smoking was politically motivated and articulated within Chinese cultures, propelling a spirit of independence and autonomy. Masculine ideals are multifaceted and influence men’s smoking locally, regionally and globally [38]. So, while direct fathering and protector and provider roles prompted the fathers to abstain in the local context many fathers did not regard their continued (light) smoking away from their family as diminishing their effectiveness as a father. In this way, the Chinese fathers who continued smoking did so outside the role of fathering, within regional and global arenas and contexts where smoking was accepted – and affirmed. According to the fathers, they believed smoking would not be harmful if they smoked in a controlled manner. This finding raises potential challenges for future smoking cessation interventions, because the current slogan to motivate Canadian fathers to quit smoking, “Don’t let your children be a target. Make your home smoke-free”, [46], while effective in the local context, might have little weight in other contexts.

### Implications for smoking cessation interventions

There are very few smoking cessation interventions in North America targeting Chinese immigrant men or more specifically fathers. Instead, tobacco control approaches have tended to be gender and culture neutral [47,48]. The current study findings confirm previous research indicating many Chinese immigrants had either quit smoking or substantially reduced their smoking [8-11]. This trend would be substantially advanced with targeted interventions.

Central to supporting these cessation efforts will be effective education to correct misinformation about the health risks of light smoking [49]. This is important because, over the last 10 years, the prevalence of light smoking subgroups in the USA have steadily increased, and now account for more than 20% of individuals who smoke [49]. Light smokers may be more receptive than previously believed to messaging about the risks of continued smoking [49]. The current study reveals two time points when Chinese Canadian men re-think their smoking: immediately after arrival in Canada, and during their partners’ pregnancies. Relevant health messages may be easily taken up by the Chinese immigrant smokers if they coincide with the smokers’ life events. Printed or online health messages can be developed in accord with the masculine ideals of Chinese Canadian men. Oliffe et al. [32] suggested three principals to guide the development of the health messages targeting men: 1) using positive messaging to promote change without amplifying stigma, guilt, shame, and blame; 2) fostering connections between masculine ideals (e.g., strength, decisiveness, resilience, autonomy) and being smoke-free; and 3) privileging the testimonials of potential end-users (e.g., fathers who smoke and want to quit). These principles might reasonably guide culture sensitive approaches that appeal to specific masculine norms such as familism and collectivism among Chinese Canadian men.

The findings from the current study are limited in that they cannot be reliably generalized to large populations of Chinese men. Although the findings indicate that the high cost of cigarettes, comprehensive smoking bans in public places and the denormalization of tobacco use in general influenced Canadian Chinese fathers to quit smoking, some men did not quit. There is a need for further research with diverse groups of Chinese men to extend our understanding of the influence gender-related factors and other social determinants on smoking patterns to inform the development of tailored cessation interventions.

## Conclusions

This description of Chinese immigrant fathers’ experiences related to tobacco reduction and cessation in Canada adds to the growing knowledge regarding gender-related factors influencing men’s smoking. Chinese immigrant men who smoke tend to quit or reduce their smoking prompted by an interplay of bi-cultural factors related to immigration, being a new father, and desires to conform to tobacco norms and fathering roles in the host country. Traditional Chinese familism complemented Canadian norms related to protecting children from secondhand smoke, and contributed to the men becoming smoke free or reducing their levels of smoking. Increased child care responsibilities and decreased networking with male friends and colleagues in Canada resulted in less time and fewer settings that facilitated tobacco use. In contrast, for those who continued to smoke at reduced levels, tobacco was perceived as a reward for fulfilling masculine provider roles under difficult circumstances. The findings have implications for the development of future smoking cessation interventions targeting Chinese Canadian immigrant smokers as well as smokers in China.
